# Delayed Diagnosis of Inborn Errors of Immunity Due to CISH Gene Mutation Presenting As Recurrent Tuberculosis

**DOI:** 10.7759/cureus.70737

**Published:** 2024-10-02

**Authors:** Adeeb Munshi, Ali Almontasheri, Raneem Arab, Majed Alshehri

**Affiliations:** 1 Adult Allergy and Clinical Immunology, King Abdulaziz Medical City, Jeddah, SAU; 2 College of Medicine, King Saud Bin Abdulaziz University for Health Sciences, Jeddah, SAU; 3 Family Medicine, King Abdullah Medical City, Jeddah, SAU

**Keywords:** cish, gene, lymphadenitis, mutation, tuberculosis

## Abstract

Tuberculosis (TB) is an infectious disease that most often affects the lungs but can be extrapulmonary, with TB lymphadenitis being the most common extrapulmonary site. The case presented here describes a 54-year-old female patient who was treated for pulmonary TB 20 years prior to this presentation, presented this time with fever, bilateral neck swelling, and unintentional weight loss of 20 kg over four months and histopathological findings of caseating granuloma of the biopsy taken from the right cervical lymph node. After investigation by the immunology team, a genetic test was done. She was found to have a multiple cytokine-inducible SH2-containing protein (CISH) genetic mutation, a discovery that is particularly significant given her history of recurrent TB.

## Introduction

Tuberculosis (TB) is an infection that primarily involves the lungs, and *Mycobacterium tuberculosis *(MTB) is the organism responsible for it [1]. The most common presentation of extrapulmonary TB is TB lymphadenitis, and TB accounts for up to 43% of peripheral lymphadenopathy in resource-limited areas [2-3]. TB is suspected in patients who show relevant clinical features and epidemiological factors, and the diagnosis is confirmed by isolating MTB through culture from body secretions, fluid, or tissues. While TB poses a significant threat to public health worldwide, the introduction of anti-TB drugs has successfully treated most of the patients. However, TB recurrence after treatment contributes significantly to the public health burden [4].

The gene multiple cytokine-inducible SH2-containing protein (CISH) has been linked to immunity, as mutations in this gene can make individuals more vulnerable to serious infections like bacteremia, TB, and severe malaria [5].

Here, we describe the case of a female patient who had been treated for pulmonary TB 20 years ago but presented again with TB lymphadenitis. Subsequent testing revealed that she had a genetic mutation in the CISH gene.

## Case presentation

A 54-year-old female housewife living in Jeddah city had pulmonary TB 20 years before her current presentation, which was diagnosed and treated in an outside hospital. At that time, she presented with a cough, shortness of breath, and fever for one month; she received a six-month course of anti-TB medications, which resulted in the resolution of her infection. She was referred from the primary care clinic to the infectious diseases (ID) clinic due to suspicion of TB lymphadenitis. She presented to the ID clinic complaining of subjective fever on and off, bilateral neck swelling, and unintentional weight loss of 20 kg over four months. She denied any cough, shortness of breath, sputum production, hemoptysis, recent upper respiratory tract infection (URTI), or swelling elsewhere in her body. She denied ingesting raw milk, exposure to animals, or travel. Her history was also negative for any back pain, joint pain, joint swelling, oral ulcers, genital ulcers, or any skin rash. Her family history was positive for spinal TB in her 17-year-old son, who was diagnosed based on a histopathological examination of the biopsy taken from the spine two years prior to his mother's presentation and received a one-year course of anti-TB. Her family history was negative for malignancy or autoimmune diseases.

Physical examination at the time of presentation showed blood pressure of 114/67 mmHg, heart rate of 65 beats per minute, respiratory rate of 18 breaths per minute, temperature of 36°C, oxygen saturation of 100% on room air, and body mass index (BMI) of 18.3 kg/m^2^. Chest and abdominal examinations were unremarkable. Lymph node examination was remarkable for multiple firm, non-tender, non-fluctuant masses in the bilateral cervical region. The largest mass was in the right cervical, measuring around 2 cm. Other systemic examinations were unremarkable.

Laboratory investigations revealed a white cell count (WBC) of 3.6x10^9^/L (4.0-11.0x10^9^/L), neutrophils of 0.82x10^9^/L (2.00-7.50x10^9^/L), C-reactive protein (CRP) 1.4 mg/L (0-5 mg/L), and erythrocyte sedimentation rate (ESR) 21 mm/hr (0-20 mm/hr). QuantiFERON TB was requested, which came out positive. HIV-1 serology (combination antigen/antibody immunoassay), Brucella IgM, and IgG all were requested, which were negative (Table 1).

**Table 1:** Laboratory investigations of the patient upon presentation WBC: white blood cell count; CRP: C-reactive protein; ESR: erythrocyte sedimentation rate; AST: aspartate aminotransferase; ALT: alanine aminotransferase; TSH: thyroid stimulating hormone; TB: tuberculosis; HIV: human immunodeficiency virus; IgM: immunoglobulin M; IgG: immunoglobulin G; NS1: nonstructural protein 1; CMV: cytomegalovirus; VCA: anti-viral capsid antigen; EBNA: Epstein-Barr nuclear antigen

Variable	Upon presentation	Reference range
WBC	3.6	4.0-11.0x10^9^/L
Neutrophils	0.82	2.00-7.50x10^9^/L
Lymphocytes	2.14	1.50-4.00x10^9^/L
Monocytes	0.23	0.20-0.80x10^9^/L
Eosinophils	0.21	0.10-0.70x10^9^/L
Basophils	0.02	0.00-0.10x10^9^/L
Hemoglobin (gm/dL)	13.3	11.5-16.5
CRP (mg/L)	1.4	0.0-5.0
ESR (mm/hr)	21	0-20
Creatinine (ummol/l)	60	50-74
AST (IU/L)	33	5-34
ALT (U/L)	16	6-28
TSH (mIU/L)	2.46	0.60-5.00
QuantiFERON TB	Positive	
HIV-1 serology (combination antigen/antibody immunoassay)	Negative	
Brucella IgM	2.3	<8 U/mL = Negative U/mL = Borderline >12 U/mL = Positive
Brucella IgG	4.3	<8 U/mL = Negative U/mL = Borderline >12 U/mL = Positive
Dengue NS1 antigen	Negative	
Dengue IgM	Negative	
Dengue IgG	Negative	
CMV quantitative PCR	Undetectable	
Anti-VCA IgM	Negative	
Anti-VCA IgG	Negative	
EBNA	Negative	

**Figure 1:**
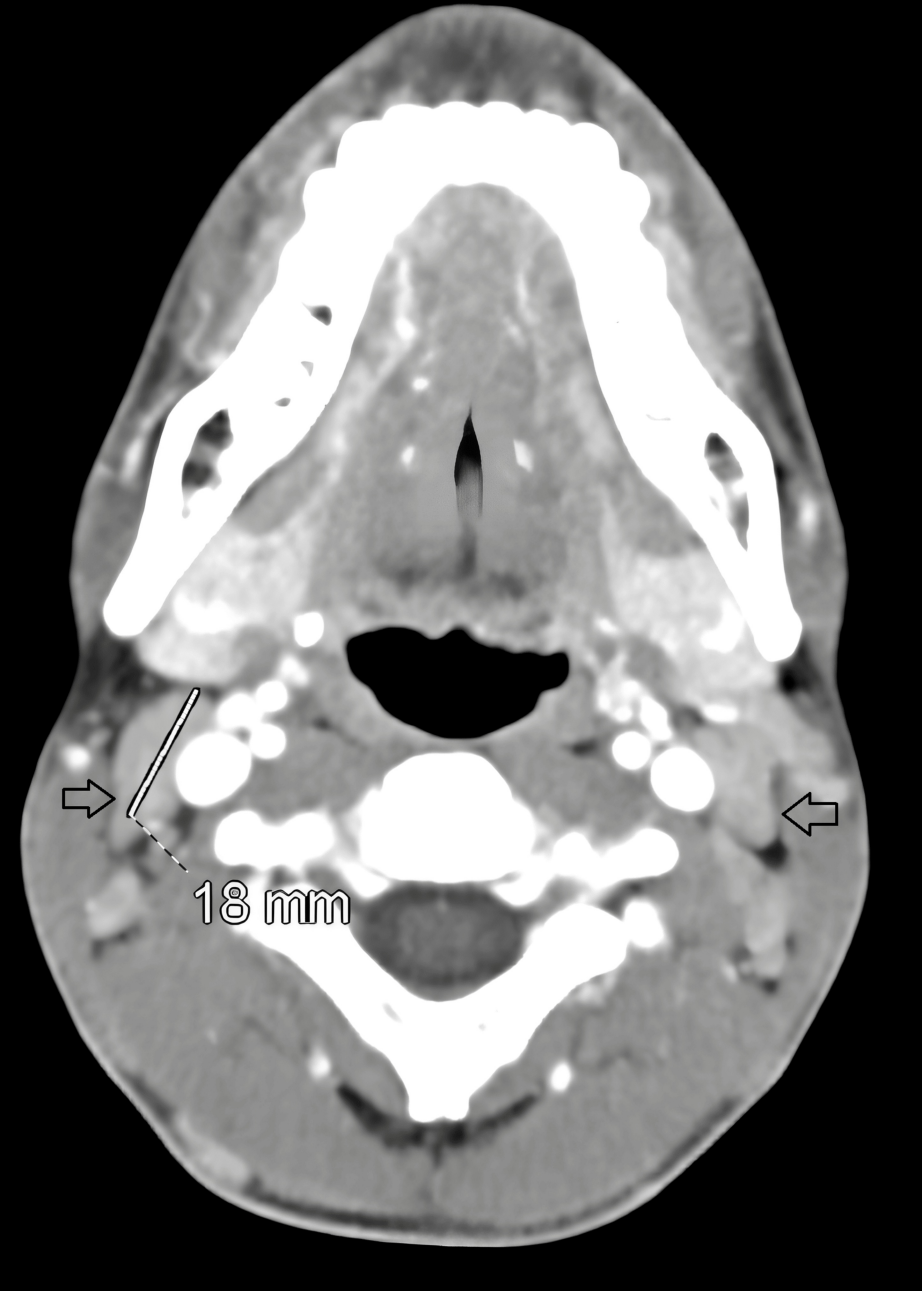
Neck and chest CT revealed multiple enlarged lymph nodes in the bilateral cervical region (black arrows), largest one in the right cervical region measuring up to 1.8 cm (white line) CT: computerized tomography

Moreover, neck, chest, abdomen, and pelvis CT were done. The findings are as follows: neck and chest CT revealed multiple enlarged lymph nodes in the bilateral cervical region, measuring up to 1.8 cm (Figure 1).

Abdominal and pelvis CT was unremarkable. The patient was referred to the hematology team to investigate the possibility of hematological malignancy due to the presence of enlarged lymph nodes. A peripheral blood smear and bone marrow biopsy were done, as shown in Table 2.

**Table 2:** Peripheral blood smear and bone marrow biopsy results AFB: acid-fast bacilli

Test	Result
Peripheral blood smear test	Leucopenia with a majority of lymphocytes seen
Bone marrow biopsy	hypocellular marrow (50% cellularity) with active hemopoiesis. No abnormal cells
Bone marrow AFB culture	Negative

A right cervical excisional lymph node biopsy was done, and the histopathology revealed the presence of caseating granuloma. However, acid-fast bacilli (AFB) smear, AFB culture, and nucleic acid amplification testing (NAAT) were negative. Also, the biopsy was negative for malignancy. So, the ID team decided to start her on first-line anti-TB medications for TB lymphadenitis. She has commenced on ethambutol 800 mg orally once daily and pyrazinamide 1000 mg orally once daily for 70 days. Isoniazid 300 mg orally once daily and rifampicin 450 mg orally once daily for nine months. The patient was seen clinically by the ID team after completion of treatment, which revealed complete resolution of his symptoms and cervical swelling.

As the patient experienced a second episode of TB, the immunology team was consulted to investigate the possibility of inborn errors of immunity as a cause of recurrent TB infection. A comprehensive set of diagnostic tests was requested, including T-cell subset analysis, which revealed reduced CD19 (B-cell) of 140.0 cells/mcL L (200.0-400.0), CD3 (T-cell) of 1070.00 cells/mcL L (1100.00-1700.00), CD3+CD4 of 510.00 cells/mcL L (700.00-1100.00), and CD16+CD56 (NK) of 64.0 cells/mcL L (200.0-400.0). The patient's CD4/CD8 ratio was found to be reduced to 0.9 L (1.00-1.50). Serum immunoglobulin levels (Ig) were requested, which showed an IgG level of 19.08 g/L (6.5-16.2 g/L), an IgM level of 1.24 g/L (0.40-3.45 g/L), and an IgA level of 4.80 g/L (0.65-4.21) (Table 3).

**Table 3:** T cell subset analysis and immunoglobulin levels of the patient CD: cluster of differentiation; NK: natural killer; Ig: immunoglobulin

Variable	Result	Reference range
CD3+ (T cells) (cells/mcL L)	1070.00	1100.00-1700.00
CD3+CD4+(T helper) (cells/mcL L)	510.00	700.00-1100.00
CD3+CD8+(T supp) (cells/mcL L)	561.00	500.00-900.00
CD19+ (B cells) (cells/mcL L)	140.00	200.00-400.00
CD10+CD56+ (NK) (cells/mcL L)	64	200.00-400.00
CD4/CD8 ratio (L)	0.91	1.00-1.50
IgA (g/L)	4.80	0.65-4.21
IgG (g/L)	21.51	6.50-16.20
IgM (g/L)	1.24	0.40-3.45

The patient was offered genetic testing, and she actively participated in her treatment by accepting and signing the consent. A blood sample withdrawn for whole exome sequencing (WES) showed that she had a stop-gain likely pathogenic mutation P.(Arg269*) in the CISH gene (Table 4). Additionally, WES was done for her affected son outside the hospital, which showed the exact genetic mutation. Segregation analysis was done for the whole family, which showed the presence of this mutation in the mother and affected son and its absence in the entire family, indicating its pathogenicity.

**Table 4:** Whole exome sequencing results of the patient CISH: Multiple Cytokine-Inducible SH2-containing protein, Arg: arginine

Gene	Variant coordinates	Amino acid change	Zygosity	Type and classification	Related disorder and mood of inheritance
CISH	NM_013324.5:c.805>T	P.(Arg269*)	Heterozygous	Stop gain Likely pathogenic variant (class 2)	Increased genetic susceptibility to infections including tuberculosis

## Discussion

Recurrent TB is defined as more than one TB episode per patient, resulting from either an exogenous new infection or relapse through endogenous reactivation of the first infection. In high TB incidence settings, the recurrence rate has been estimated to be 4.10 (95% CI 2.67 to 6.28) per 100 person-years among patients followed up for an average of 2.3 years after cure [4]. Recurrence has been linked to suboptimal treatment, depressed immunity, and probability of exposure to the agent [6]. Our patient, who had recurrent MTB infection, has been found to have an infected family member with TB; later, her WES showed a CISH genetic variant, which increased her susceptibility to TB.

The challenging part in diagnosing TB lymphadenitis is its nonspecific presentation, which could overlap with other infectious and non-infectious differential diagnoses. The gold standard is culture; however, the results could be delayed up to 3-4 weeks [7].

While the AFB stain can be a valuable tool in diagnosing mycobacterial disease, its limitations must be noted. Studies have shown poor sensitivity, ranging from 5% to 38%, underscoring the need for a comprehensive diagnostic approach [8-9].

When AFB and culture fail to provide a precise diagnosis, histopathological examination can be valuable. It can reveal findings that support TB lymphadenitis, such as caseating or non-caseating granulomas and nonspecific lymphadenitis [8].

The polymerase chain reaction (PCR) method, a key player in the diagnosis of TB, offers a significant advantage in terms of time. With its high sensitivity and specificity, PCR can yield results within 24 to 48 hours, providing a swift and accurate diagnosis [10-11].

Our patient showed typical symptoms of TB lymphadenitis, such as fever, weight loss, and multiple firm, non-tender masses in the neck area on both sides. The diagnosis was confirmed by finding caseating granuloma in the biopsy of the right cervical lymph node. The patient has multiple warning signs of inborn errors of immunity, including family history and more than one deep-seated infection (recurrence of TB infection despite that she received a complete course of treatment) in an immunocompetent patient, which prompted the immunology team to investigate [12]. Their comprehensive approach, including WES, revealed a CISH gene mutation, the same mutation found in her son, confirming the pathogenicity of the diagnosis. Additionally, segregation analysis was done for the whole family, which showed the presence of this mutation in the mother and affected son and its absence in the entire unaffected family, indicating its pathogenicity.

The traditional regimen for treatment of TB lymphadenitis consists of four drugs: isoniazid, rifampin, pyrazinamide, and ethambutol, given for two months, followed by a continuation phase where isoniazid and rifampin are continued for four months [1]. Our patient received the traditional intensive phase regimen, four drugs phase, for two months; the continuation phase, two drugs phase, was extended for seven months, so she received a total of nine months of treatment, which was longer than the standard treatment period of TB lymphadenitis (the standard duration for TB lymphadenitis is six months) given her previous episode of TB infection [1].

The CISH gene produces the CIS protein, which belongs to the suppressors of the cytokine signaling (SOCS) family and is an essential negative regulator for inflammatory signaling, Jak-STAT prolactin, and interleukin-2 signaling pathways. The mechanism behind CISH's contribution to infections could be related to increased inhibition of inflammatory cytokines. Increased CISH gene activity will exacerbate cytokine signaling pathways' inhibition, thus increasing infectious susceptibility [5].

A case-control study conducted in China concluded that specific CISH genotypes such as rs414171 TT and rs809451 CC are possible risk factors for TB infection [13]. Another study published in 2016 estimated that the overall risk of IDs among persons carrying the variant CISH alleles is to increase by at least 18%. It also stated that single nucleotide polymorphisms (SNPs) in the CISH gene are associated with increased susceptibility to various infections, emphasizing the role of negative regulators of cytokine signaling in immunity against various infections [14]. On the other hand, a case-control paper published in Iran found that CISH rs414171 and rs6768300 variants might be associated with protection from pulmonary TB [15].

## Conclusions

The rare case presented in this study showed that a patient with recurrent MTB infections had a CISH genetic mutation, which increased her susceptibility to MTB infections. The patient exhibited typical symptoms of TB lymphadenitis and was treated with extended anti-TB therapy, leading to a complete resolution in her clinical status. Our case highlights the importance of investigating underlying immunodeficiency in patients who were previously thought to be immunocompetent but experience recurring mycobacterial TB infections.
